# Measuring the frequency of emotions—validation of the Scale of Positive and Negative Experience (SPANE) in Germany

**DOI:** 10.1371/journal.pone.0171288

**Published:** 2017-02-08

**Authors:** Tobias Rahm, Elke Heise, Mirijam Schuldt

**Affiliations:** Institut für Pädagogische Psychologie, Technische Universität Braunschweig, Braunschweig, Germany; IRCCS Istituto Auxologico Italiano, ITALY

## Abstract

The Scale of Positive and Negative Experience (SPANE) measures the frequency of positive and negative affect. It consists of two subscales, one for positive and one for negative affect with six adjectives each and assesses a broad range of emotions. In this study, the psychometric properties of the German version of the scale were explored with reliability and confirmatory factorial analysis by using a German sample of *N* = 498. To examine the convergent validity of the SPANE we correlated its subscales with other well-being and happiness measures. Retest-reliabilities were tested after four weeks, taking into account the occurrence of emotionally significant life events. The scale was also applied to evaluate a training of subjective well-being and in a study on text comprehensibility. The results confirmed the postulated two-factor structure of the SPANE and showed good psychometric properties and convergent validity. In conclusion, the German version of the scale behaves in accordance with the original scale and may be used in future studies of well-being.

## Introduction

One of the aims of Positive Psychology is to analyze the conditions and consequences of well-being. High subjective well-being (SWB) is commonly defined as frequent positive affect, infrequent negative affect, and a high level of satisfaction with life in general [1]. Various scales have been developed to assess positive and negative affect. One of the most widespread, the Positive and Negative Affect Scale (PANAS) [2], is not entirely suitable for the measurement of subjective well-being because of its focus on the intensity (not the frequency) of specific (instead of general) emotions. Diener et al. (2010) therefore developed the Scale of Positive and Negative Experience (SPANE) consisting of two subscales. SPANE-P contains three general positive items and three specific positive items, SPANE-N contains three general negative and three specific negative items [3]. In combination with a scale for life satisfaction (e.g. Satisfaction with Life Scale–SWLS [4]), the SPANE allows the measurement of SWB according to the above definition. For more details on background and reasons for the development of the SPANE see the original study of Diener et al. (2010) or the validation of the Chinese version by Li et al. (2013) [3,5]. So far the homepage of Ed Diener provides the SPANE in nine languages (Arabic, French, German, Hindi, Italian, Iranian, Japanese, Polish and Turkish) and we know of validation studies for the versions in Chinese (Li et al. 2013), Japanese (Sumi 2014), Portuguese (Silva and Caetano 2011) and Turkish (Telef 2015) [5–8]. Jovanović (2015) found that the Serbian version of the SPANE used in schools better predicted well-being than the PANAS [9]. With this study we add a validated German version of the SPANE.

Various studies report positive outcomes of high well-being. In an extensive meta-analysis, Lyubomirsky et al. (2005) found that people with higher SWB are healthier, show more adaptive health behavior and are more productive at work [10]. Diener (2009) points out that after conducting numerous and even large scale studies on SWB in the past decades, we now need to ask more detailed questions and find out if effects are due to changes in life satisfaction, positive emotions or negative emotions and how these constructs interact with each other [11]. Therefore he requires studies that take into account all three subdomains of SWB using instruments that differentiate between them. As we know that high SWB is desirable in many areas of people’s lives and the functioning of organizations, one central question is how to sustainably increase SWB. Areas of special interest are for example health promotion, human resources and organizational development, educational systems, and also politics (e.g. shown in the OECD Guidelines on Measuring Subjective Well-being 2013, where the English version of the SPANE is included) [12]. As educational psychologists we focus on teaching and learning processes. In Germany, as in many other countries, teachers’ health problems and their negative consequences, such as decreasing work quality or high work-absence periods, have been an ongoing topic. Increasing teachers’ subjective well-being might work as health promotion as well as enhance the quality of work. Taking into account that happiness seems to be contagious (e.g. social network study by Fowler and Christakis 2008), even students might benefit from higher teachers’ SWB not only by the increase of teachers’ health and work quality but also by an increase of students’ positive emotions [13]. In this context, a valid measurement of SWB is important to evaluate the change and its sustainability. First results from SWB trainings with teacher students (Rahm and Heise 2015) and non specific target groups (Blickhan 2015) are promising [14–15].

To validate the German version of the SPANE, the English version was translated and re-translated. We compiled a pen and paper version and an online version of the questionnaire and recruited participants in lectures and via e-mail. In order to test convergent validity, we enclosed other measures of well-being.

In a series of studies Watson and Walker (1996) found trait affect scales–especially the PANAS scales–to be substantially stable even across extended time spans [16]. On the other hand there is variance left to be explained and we also have evidence that various interventions sustainably affect positive and negative emotions (for an extensive example see the meditation/emotion training developed and evaluated by Kemeney et al. 2012 [17]). Therefore, in addition to measuring the retest-reliability after one month we were interested in the SPANE’s sensitivity to significant life events and training interventions. We expected positive life events, such as a marriage, and negative life events, such as a serious illness of a close person, as well as positive activity interventions (Shin and Lyubomirsky, 2016) to have an impact on the frequency of positive and negative emotions [18]. Therefore, higher differences in the SPANE scales from Time 1 to Time 2 should occur if significant life events were reported or training interventions were carried out.

## Materials and methods

### Ethics statement

Participants were informed that participation was voluntary and could be cancelled at any time without any disadvantages. They were also informed that their data would be used for scientific purposes only. The survey was carried out with adult volunteers. Data were analyzed in aggregated and completely anonymous form. The study was approved by the Ethic Committee of Faculty 2 of the Technische Universität Braunschweig (FV-2016-07).

### Translation process

First, the English version of the SPANE was translated into German by the authors. Minor discrepancies were discussed and a preliminary version was created. This version was translated back into English by a bilingual psychologist. Again, small discrepancies were discussed and the final version was agreed upon. The translation of “angry” provoked the strongest controversy concerning the alternatives “ärgerlich”, which seems to be rather passive and is closer to “displeased”, and “wütend” which is more extraverted and closer to “rage”. As the other two specific negative adjectives (“afraid” and “sad”) are more introverted we decided on “wütend” as the best option.

### Sample

Initially, 512 participants were assessed. 14 of them did not answer all 12 items of the SPANE and had to be excluded from further analysis so that the total size of the sample was *N* = 498. About one half of the sample was recruited in lectures at the University of Braunschweig (*n* = 264). These participants were given time to fill in the pen and paper questionnaire right away. The other half was recruited via mailing lists and the social media platform Facebook (*n* = 234). These participants completed the online version of the questionnaire. 374 participants were female (75%), 338 were students (68%). About 89% had passed the “Abitur” which is the highest form of graduation from school in Germany. 47% were aged between 20 and 29 years. All participants completed a demographic questionnaire and the measures described later. Participants could voluntarily build a unique code and give their e-mail address for the 1-month follow-up. The paper version had an extra sheet for the e-mail address, which was separated from the questionnaire before the data were transferred. In the online version the e-mail addresses were separated from the data file right after downloading. After one month we contacted all collected e-mail addresses and invited participants to fill in the online questionnaire again which was done by 105 participants.

### Data analysis

A confirmatory factor analysis (CFA) was conducted to test the two factor structure found in previous studies [3,5–7]. Convergent validity was tested using other measures of happiness, SWB, and life satisfaction. Additionally, we tested retest-reliability after one month taking into account reported significant life events within this time period. Finally, the SPANE was applied to evaluate a training of subjective well-being. All analyses were computed with SPSS and AMOS 23.

### Measures

The following measures were applied to assess convergent validity.

#### SPANE–Scale Of Positive And Negative Experience [3]

The SPANE measures the frequency of positive (SPANE-P) and negative (SPANE-N) emotions with two 6-item-subscales. Both subscales consist of three adjectives describing rather general feelings (e.g. pleasant / unpleasant) and three adjectives describing rather specific feelings (e.g. joyful / sad). Participants had to indicate how often they had felt this way during the last four weeks on a 5-point-scale ranged from 1 (very rarely or never) to 5 (very often or always). Scores per subscale are added so that both subscales range from 6 to 30. To reduce data complexity, Diener et al. (2010) also combined both scales by subtracting the negative score from the positive score and named this balanced score SPANE-B. As will be discussed later, we do not follow the idea of this complexity reduction but we will nevertheless report data on SPANE-B for comparison purposes. The German version of the scale can be found online in S1 File.

#### SWLS–Satisfaction With Life Scale [4,19]

The SWLS assesses the satisfaction with life in general. It consists of five items (e.g. “I am satisfied with my life”) which have to be rated on a 7-point scale ranging from 1 (strongly disagree) to 7 (strongly agree). We used a German version validated by Glaesmer et al. (2011) [19].

#### PANAS–Positive And Negative Affect Schedule [2,20]

The PANAS measures the intensity of positive (PANAS-P) and negative (PANAS-N) emotions with a 10-item-scale each. Participants have to indicate for all 20 adjectives (e.g. proud, attentive / upset, ashamed) to what extent they had felt this way during a given time period (e.g. past few weeks) on a 5-point-scale ranging from 1 (very slightly or not at all) to 5 (extremely). Scores per subscale are added so that both subscales range from 10 to 50. We used a German version validated by Krohne et al. (1998) [20].

#### SHS–Subjective Happiness Scale [21–22]

The SHS assesses global subjective happiness with four items. Respondents are asked to rate both their absolute happiness and their happiness relative to others. The last two items describe very happy and unhappy individuals, respectively, and ask respondents to what extent the descriptions resemble them. The response formats are 7-point Likert scales. We used a German version validated by Swami et al. (2009) [22].

#### HSWBS–Habituelle Subjektive Wohlbefindensskala [23]

The HSWBS is an established German instrument to measure subjective well-being. It consists of two subscales: HSWBS-LZ asks for the general satisfaction with life while HSWBS-S assesses the emotional component of subjective well-being (presence of positive and absence of negative emotions). We only used the subscale HSWBS-S which consists of 6 items. Respondents have to rate each item on a 6-point Likert scale.

#### Additional information

Furthermore, participants were asked for information on age, gender, level of education and occupation or study course. The retest-questionnaire additionally asked if participants had experienced any significant positive or negative life events during the last four weeks and to specify these. The entire questionnaire consisted of 52 items and could be completed within less than 10 minutes.

## Results

### Factorial validity

We tested two models with confirmatory factor analysis. As can be seen in Table 1, the two-factor structure fits the data better than the unidimensional model. Following Schermelleh-Engel et al. (2003) NFI and GFI indicate an acceptable fit (≥ .90), as well as CFI (≥ .95) and RMSEA (≤ .08) while AGFI indicates a good fit (≥ .90) [24]. The AIC is smaller in the two-factor structure, showing that this model fits the data better.

**Table 1:** Goodness of fit statistics for tests of factorial validity.

	Chi^2^	df	chi^2^/df	CFI	GFI	AGFI	RMSEA	NFI	AIC
**Single factor**	434.642	54	8.048	.865	.839	.767	.119	.850	482.642
**Two correlated factors**	177.153	53	3.342	.956	.944	.918	.069	.939	227.153

#### Descriptive analysis and internal consistency

Table 2 shows the means, standard deviations and internal consistencies for the German, English, and Chinese versions [3,5]. As can be seen, the mean of SPANE-P of the German version is close to the value of the American sample while the one for SPANE-N is close to the Chinese sample. The standard deviations are between the ones from the American and the Chinese sample. Internal consistencies for both SPANE-P and SPANE-N are good. Table 3 also shows that only one item (namely “angry”) would increase Cronbach’s alpha if deleted.

**Table 2:** Means, standard deviations, and internal consistencies of SPANE versions.

	German (*N* = 498)			English (*N* = 681)			Chinese (*N* = 21322)		
	*M*	*SD*	*α*	*M*	*SD*	*α*	*M*	*SD*	*α*
**SPANE-P**	22.25	3.88	.88	22.05	3.73	.87	21.03	4.63	.92
**SPANE-N**	14.31	4.24	.82	15.36	3.95	.81	14.37	4.84	.91

**Table 3:** Item communalities and variation in internal consistency.

Item (English)	Item (German)	Communalities	Corrected item-total correlation	Cronbach’s alpha, if item deleted
**SPANE-P (Cronbach’s alpha = .88)**				
**positive**	positiv	.56	.74	.85
**good**	gut	.50	.68	.86
**pleasant**	angenehm	.38	.59	.87
**happy**	glücklich	.58	.74	.84
**joyful**	von Freude erfüllt	.46	.65	.86
**contented**	zufrieden	.51	.71	.85
**SPANE-N (Cronbach’s alpha = .82)**				
**negative**	negativ	.59	.73	.76
**bad**	schlecht	.55	.69	.77
**unpleasant**	unangenehm	.35	.57	.79
**sad**	traurig	.48	.67	.77
**afraid**	ängstlich	.26	.49	.81
**angry**	wütend	.17	.39	.83

#### Retest-reliability

One month after the main data collection we invited all participants who had given their consent to complete the SPANE again online. As can be seen in Table 4, the retest-reliabilities of *r* = .62 for SPANE-P and *r* = .64 for SPANE-N are good. To further assess the sensitivity of the measure we asked if participants had experienced significant negative life events such as illness, death of a close person or breakup of a relationship or significant positive events such as marriage, birth of a child or begin of a relationship during this time period. The results are shown in Table 4. For comparison purposes we also included the values for SPANE-B.

**Table 4:** Retest-reliability of the German SPANE.

SPANE-P	*N*_*1*_	*M*_*1*_	*SD*_*1*_	*N*_*2*_	*M*_*2*_	*SD*_*2*_	*r*
sample total	498	22.25	3.88	105	22.37	4.01	.62**
without significant life events	64	23.41	3.18	64	23.44	3.29	.70**
with significant life events	41	22.37	3.63	41	20.71	4.49	.53**
**SPANE-N**	***N***_***1***_	***M***_***1***_	***SD***_***1***_	***N***_***2***_	***M***_***2***_	***SD***_***2***_	***r***
sample total	498	14.31	4.24	105	13.56	4.27	.63**
without significant life events	64	13.17	4.07	64	12.39	3.76	.79**
with significant life events	41	14.61	3.76	41	15.39	4.42	.38*
**SPANE-B**	***N***_***1***_	***M***_***1***_	***SD***_***1***_	***N***_***2***_	***M***_***2***_	***SD***_***2***_	***r***
sample total	498	7.93	7.37	105	8.81	7.66	.67**
without significant life events	64	10.23	6.53	64	11.05	6.39	.80**
with significant life events	41	7.76	6.34	41	5.32	8.24	.48*

** *p* < .001;

*p < .05.

#### Convergent validity

To assess construct validity we correlated the SPANE with other measures of subjective well-being which were described above. The subscales SPANE-P and SPANE-N are negatively correlated with *r* = -.65 which is close to the correlation of *r* = -.60 reported in the original study by Diener et al. (2010). The correlations between both SPANE and PANAS and between SPANE and SWLS are quite similar to those within the American sample. SHS displays stronger correlations within the German sample for both subscales and SPANE-B (cf. Table 5).

**Table 5:** Construct validity: Convergence with other relevant scales.

	German Sample			American Sample [3]		
	SPANE-P	SPANE-N	SPANE-B	SPANE-P	SPANE-N	SPANE-B
**SPANE-N**	-.65**			-.60**		
**SPANE-B**	.90**	-.92**		n.a.	n.a.	
**PANAS-PA**	.61**	-.46**	.58**	.61**	-.46**	.58**
**PANAS-NA**	-.50**	.77**	-.70**	-.46**	.70**	-.65**
**SHS**	.66**	-.60**	.70**	.56**	-.48**	.58**
**SWLS**	.61**	-.50**	.61**	.58**	-.46**	.57**
**HSWBS-S**	.68**	-.55**	.67**	n.a.	n.a.	n.a.

** *p* < .001;

n.a. not available

### Further validation

#### Training of subjective well-being

We also used the SPANE to evaluate a four-week-training of subjective well-being for future teachers with one training day every two weeks (Rahm and Heise 2015) [14]. Psycho-educational topics included happiness and well-being, positive emotions, self-efficacy, attributions, gratitude, mindfulness, good deeds and flow as well as rumination and social comparisons. Participants were asked to perform the exercise *Three Good Things* (Seligman et al. 2005) [25], writing down three positive experiences of the day as well as their contribution to these experiences every evening of the four weeks training period. Additionally, they planned three activities (savoring experience, gratitude visit and good deed day) to further develop well-being and chose one of these activities to realize. The main focus of the training was to increase positive emotions while also trying to reduce negative emotions and increase life satisfaction. The training was evaluated with the German SPANE and other instruments assessing subjective well-being which were applied as pen and paper questionnaires before and after the training and as online questionnaires at one-, three- and six-month-follow-up. The training was offered as a required elective course within the teacher education program at Braunschweig University. The control group was recruited within other courses, via mailing lists and a social media platform. Control participants did not receive any intervention and filled in all questionnaires online.

As shown in Table 6 we found significant increases in self-reported positive experiences (SPANE-P) with small to medium effect sizes from pre-test to post-test and all follow-ups in the training group. Neither the changes in the control group nor those in self-reported negative experiences (SPANE-N) in both groups were significant. For SPANE-P also the group x time interaction showed significantly greater changes in the training group than in the control group but only from pre to post (*t*_(133)_ = 3.24; *p* < .01; *d* = 0.59).

**Table 6:** SWB-Training-Evaluation with the SPANE.

**SPANE-P**									
**t1**	**t2**		***N***	***Mean***_***t1***_	***SD***_***t1***_	***Mean***_***t2***_	***SD***_***t2***_	***T***	***d***
**pre**	**post**	**TG**	45	21.13	4.17	23.38	3.11	5.26	0.58*
		**CG**	90	22.59	3.56	22.77	3.76	.45	0.05
**pre**	**1Mfu**	**TG**	34	21.97	3.96	23.06	3.70	2.09	0.28*
		**CG**	73	21.95	4.08	22.27	3.99	.76	0.08
**pre**	**3Mfu**	**TG**	40	21.38	4.32	22.85	4.23	2.18	0.35*
		**CG**	71	22.01	3.34	22.31	3.62	.67	0.09
**pre**	**6Mfu**	**TG**	35	21.26	4.45	22.54	4.42	2.25	0.29*
		**CG**	90	22.36	3.62	22.56	4.26	.48	0.05
**SPANE-N**									
**t1**	**t2**		***N***	***Mean***_***t1***_	***SD***_***t1***_	***Mean***_***t2***_	***SD***_***t2***_	***T***	***d***
**pre**	**post**	**TG**	45	14.33	4.62	13.38	4.53	-1.89	-0.21
		**CG**	90	13.84	4.34	13.58	4.21	-.59	-0.06
**pre**	**1Mfu**	**TG**	34	13.85	4.77	13.32	4.60	-.92	-0.11
		**CG**	74	14.09	4.53	13.36	4.14	-1.76	-0.17
**pre**	**3Mfu**	**TG**	39	14.41	4.93	13.56	5.20	-1.08	-0.17
		**CG**	71	14.00	4.29	13.41	3.89	-1.33	-0.14
**pre**	**6Mfu**	**TG**	34	14.21	4.87	14.32	5.19	.18	0.02
		**CG**	90	13.88	4.09	13.52	4.17	-.75	-0.09

* *p* < .05, pre: pre-test, post: post-test, 1Mfu: one-month-follow-up, 3Mfu: three-month-follow-up, 6Mfu: six-month-follow-up, TG: training group, CG: control group, *d*: effect size Cohen’s *d*.

## Discussion

The German SPANE may be considered an economical short scale that validly measures the frequency of positive and negative affect with good internal consistencies. The two-factor structure fits the data better than a single-factor model and both scales showed high correlations with other relevant scales measuring convergent constructs. The results of our analyses are comparable to those reported for the original SPANE as well as for the Chinese version [3,5]. The scale also proved to be sensitive to emotionally significant life events and complex interventions such as a training to increase subjective well-being. The evaluated training aimed at the increase of positive emotions. From pre to post-testing and all follow-ups we found significant small to medium effect sizes with SPANE-P and only a small but non-significant effect with SPANE-N from pre to post. These results can thus be interpreted as further evidence of the validity of the SPANE. Friedrich (2015) used the German SPANE to validate his questionnaire measuring text comprehensibility [26]. The original instruction of the SPANE, however, was reformulated in this study in order to assess the intensity of positive and negative emotions while reading texts of high vs. low comprehensibility. The results showed that the subscale “perceived comprehensibility” was positively correlated with the intensity of positive emotions (*r* = .41) and negatively correlated with negative emotions (*r* = -.32) during reading. This result not only confirms the validity of the scale but also encourages applying the SPANE to various fields of research.

As mentioned above, one main reason for the development of the SPANE by Diener and colleagues was to measure the frequency instead of the intensity of positive emotions [3]. This aim could also have been reached by changing the instruction of the PANAS or other equivalent instruments. Therefore we think that the main advantage of the SPANE item list is the wider range of emotional states covered due to the well-developed mixture of specific and general emotions.

Diener et al. (2010) computed the balance score SPANE-B by subtracting SPANE-N from SPANE-P [3]. Replication studies, e.g. from China or Portugal, followed this procedure [5,7]. Of course the idea of having only one value to report and discuss is intriguing. The problem that occurs, however, is that we cannot be sure how to interpret this resulting value. A principal axis factor analysis by Silva and Caetano (2013) on the complete item list of the SPANE yielded two distinct factors [7]. CFAs in our and other studies [5–7] also showed the two-dimensional structure of the SPANE. Therefore, the subscales need to be interpreted as two dimensions and not as two poles of one continuum. Thus, subtracting one value from the other leads to a loss of information. For example, a SPANE-B value of, for instance, 6 would tell us that this individual reported more positive than negative affect (Diener and colleagues report a mean of 6.69 with *SD* = 6.88) [3]. We cannot differentiate if he or she had high rates of both positive and negative feelings (say 26 and 20) or felt almost nothing at all (say 8 and 2). In these two cases, however, totally different interventions would be needed to become happier and more satisfied with life. Therefore we do not recommend computing and interpreting SPANE-B.

Our findings are limited to the specifications of our sample. Although we were also able to recruit a number of non-student participants, the sample was not well-balanced with respect to participants’ sex, age and educational level. Therefore, a replication with a more heterogeneous sample seems desirable. Also future research on discriminant validity of the German SPANE may complement our findings on convergent validity. Another methodological issue is the translation of the item “angry” into “wütend” which showed a factor loading less than .50 and, if deleted, would increase the internal consistency of the scale. Since the German adjective “wütend” refers to a stronger feeling than, for instance, the adjective “ärgerlich”, our translation may have led to a higher item difficulty compared to the original version. This could also explain the lower means of SPANE-N as compared to the American sample [3]. An approach to deal with this difficulty could be to develop a short form of the SPANE consisting of the six more general items, as Diener et al. suggested [3].

We also agree with Diener et al.’s suggestion that in a next validation step the SPANE should be correlated with non-self report measures, such as ratings by family members, friends or experts [3]. An even more extensive way to assess subjective well-being is the so called Experience-Sampling Methodology [1], where emotions are documented several times a day via electronic devices or smartphone applications. Correlating ESM data with the SPANE might provide deeper insight into the frequency and intensity of emotions and into processes of accumulating positive and negative feelings over time.

Watson et al. (1988) correlated positive affect and negative affect with many other constructs such as social activity or perceived stress [2]. A question for further research is whether these correlations can also be found with the SPANE. The differences between a frequency instruction and an intensity instruction should also be systematically analyzed. Findings could lead to better tailored interventions to enhance subjective well-being. Using the SPANE to evaluate such interventions on a longer term and collect more data on sensitivity may also be a fruitful contribution to the field of Positive Psychology.

## Supporting information

S1 Dataset SPANE German Validation. (SAV)

S2 Dataset SPANE German Training Evaluation. (SAV)

S1 File SPANE German Scale. (DOCX)
