# Supplementary material for: Measuring the frequency of emotions—validation of the Scale of Positive and Negative Experience (SPANE) in Germany
Source: PLoS One. 2017 Feb 8;12(2):e0171288. doi: 10.1371/journal.pone.0171288 (PMC5298234; doi:10.1371/journal.pone.0171288)
Supplement: S1 File — (DOCX) [file pone.0171288.s003.docx]

**Validated German version of the Scale of Positive and Negative Experiences (SPANE)**

Reference:

Rahm, T., Heise, E., Schuldt, M. (2016). Measuring the Frequency of Emotions - Validation of the Scale of Positive and Negative Experience (SPANE) in Germany. Submitted to PLOS ONE.

Authors:

Tobias Rahm*, Elke Heise, Mirijam Schuldt

Technische Universität Braunschweig

Institut für Pädagogische Psychologie

t.rahm@tu-braunschweig.de

** corresponding author*

| **Bitte denken Sie an das, was Sie in den *letzten 4 Wochen* getan und erlebt haben. Anschließend kreuzen Sie bitte in der folgenden Liste an, wie häufig Sie sich so gefühlt haben.** |
| --- |

|  | sehr selten oder nie | selten | gelegentlich | oft | sehr oft  oder immer |
| --- | --- | --- | --- | --- | --- |
| positiv | □ | □ | □ | □ | □ |
| negativ | □ | □ | □ | □ | □ |
| gut | □ | □ | □ | □ | □ |
| schlecht | □ | □ | □ | □ | □ |
| angenehm | □ | □ | □ | □ | □ |
| unangenehm | □ | □ | □ | □ | □ |
| glücklich | □ | □ | □ | □ | □ |
| traurig | □ | □ | □ | □ | □ |
| von Freude erfüllt | □ | □ | □ | □ | □ |
| ängstlich | □ | □ | □ | □ | □ |
| zufrieden | □ | □ | □ | □ | □ |
| wütend | □ | □ | □ | □ | □ |
